# Individual Variability, Statistics, and the Resilience of Nervous Systems of Crabs and Humans to Temperature and Other Perturbations

**DOI:** 10.1523/ENEURO.0425-23.2023

**Published:** 2023-11-14

**Authors:** Eve Marder

**Affiliations:** Volen Center and Biology Department, Brandeis University, Waltham, MA 02454

**Keywords:** climate change, crustaceans, epilepsy, MS, temperature

Every so often, I conclude that life is not possible. It is not uncommon for me to walk out of a seminar about the pathways and dynamics of biochemical signaling or the structure of biological molecules to conclude that the complexities of life processes defy imagination. Our ability to maintain a sense of wonder about the mysteries of biological mechanisms is what drives us as scientists, as otherwise wresting new insights from the recalcitrant world of interacting pathways would be too frustrating. So all successful biologists must, paradoxically, see both the proverbial forest and their trees, and recognize both the elegant simplicity and the confounds characteristic of living organisms.

That sense of mystery and wonder is somewhat at odds with our common sense. It is common sense that is now too often lost, as we grapple with new technologies and large datasets in our science. As scientists, today, we must balance our common sense with our growing reliance on big data to extract the new insights about biological systems that will allow us and the planet we steward to survive into the future. I am concerned about results that are not readily observable in raw data, but are only visible after fairly complex analysis methods are used to extract features of those data, especially if those methods have (as they usually do) all kinds of assumptions built into their analysis pipelines. I do not mean to argue against the use of complex analysis methods, but remain leery when I cannot see the results directly in raw data.

## Individual Variability and Failure of Averaging

Even young children know that each human being is an individual, and even young children know that humans have features and attributes that distinguish them from dogs, cats, elephants, and cockroaches. Children also can recognize the changes that occur with age in humans, or the difference between beagles and golden retrievers. Children know that both golden retrievers and beagles normally have four legs, but that the loss of a leg does not change the identification of a beagle as a beagle. So children, as part of their learning about the world, understand the salience of individual differences in humans and dogs, and they intuitively learn which attributes they can generalize to all individuals of a class, although racism and sexism can be seen as a consequence of educating the young with incorrect or inappropriate generalizations of attributes.

In the early days of circuit study, a single, clear-cut example of a connection between two identified neurons was assumed to generalize, and I suspect that some of the connectivity diagrams created 60 or 70 years ago had some results from a single or two observations. At the same time, because behavioral data are inherently noisy, the importance of statistics for evaluating the reliability of observations was understood to be crucial to establish the reliability of behavioral observations, followed by the adoption of statistics in all fields of biology, and their continued evolution (Bernard, 2019). And I am sure that there are generations of neuroscientists who drew comfort in statistics to assure themselves that their results were “real” despite the variance in their data.

**Figure 1.**
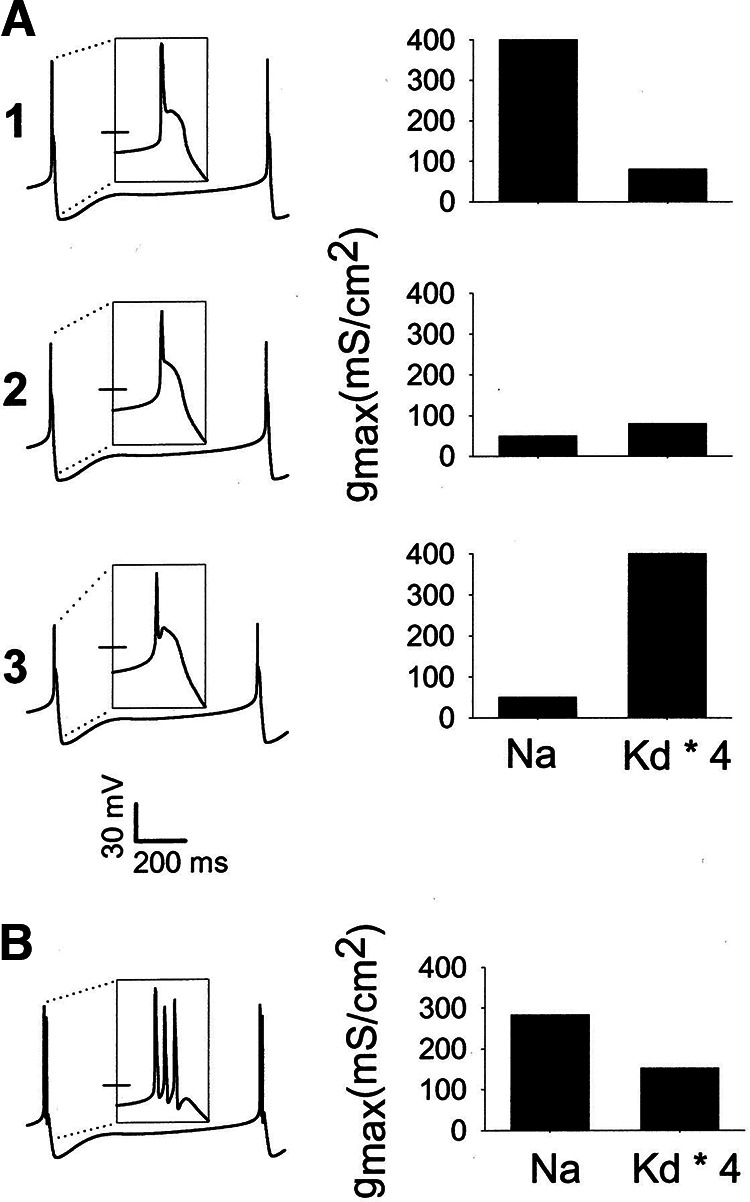
Failure of averaging in a conductance-based model neuron. ***A***, Left panels, Voltage traces for three one-spike bursters. The insets are 50 ms in width and have tick marks at −30 mV. Right panels, The values of the Na^+^ and four times the delayed-rectifier (Kd) maximal conductances. ***B***, Neuron generated from the average conductances of all the one-spike bursters. From Golowasch et al. (2002).

Nonetheless, calculating and comparing means carries risks. An example of this “failure of averaging” is seen in Figure 1. In this computational study (Golowasch et al., 2002), 164 spontaneously active model neurons with similar behavior characterized by a single action potential followed by a sustained plateau (single-spike bursters) were identified from among a larger population of 2000 models. These model neurons are examples of multiple solutions, or degenerate behavior, such as is often found in biological systems. While the voltage waveforms shown on the left were quite similar, the maximal conductances of the Na^+^ current and delayed rectifier K^+^ currents varied considerably across the models. Similar results were seen in experimental data (Swensen and Bean, 2003, 2005).

One potential danger of averaging data is illustrated in Figure 1*B*. Here, the data from the 164 single spike bursters were averaged, but when a model was built from those averaged data it was not a single spike burster, but rather fired three spikes/burst. Therefore, the model constructed using the mean data did not accurately reflect the characteristics of any of the individuals used to calculate the means!

Obviously, means often do reflect the properties of the individuals whose data were used to calculate the means. Nonetheless, if there are two (or more) distinct populations of individuals that form the greater group, wildly misleading data may result. For example, if fMRI studies reveal two brain areas of interest in mean data, it is possible that a subset of individuals are “lighting up” one area, and a different subset of individuals are activating a different area. An obvious illustration of this principle can be seen if one calculates the mean number of testicles in a population of 100 undergraduates in a classroom, and fails to recognize that male and female students should not be pooled for this purpose.

While the previous example is worthy of a chuckle, it is important to realize that as we face classification of neurons or individual animals, we may not know the essential features salient for that classification, and therefore might erroneously conclude that there is no relationship between an environmental stimulus or a therapeutic agent, if those relationships are only present in a subset of individuals, and data from distinct populations are erroneously pooled. This is potentially concerning when cells are clustered or classified by transcriptional measures alone, as there can be numerous influences that alter transcription, potentially creating circular arguments in our understanding of circuits.

## The Path from Individual Variability to Climate Change

Motivated partially by the findings from models that similar outputs could result from variable sets of neuron and circuit parameters (Goldman et al., 2001; Prinz et al., 2004; Alonso and Marder, 2019), we set out to measure a number of conductances using voltage clamp and ion channel gene expression in single identified neurons (Schulz et al., 2006, 2007; Goaillard et al., 2009; Tobin et al., 2009). These studies revealed a 2- to 6-fold range of any given parameter across animals, consistent with studies in other preparations (Swensen and Bean, 2003, 2005; Norris et al., 2011; Roffman et al., 2012; Wenning et al., 2014, 2018) and in models (Taylor et al., 2006, 2009).

This immediately raises the question whether animals with considerably different sets of network parameters can respond robustly and reliably to environmental perturbations. For this reason, we looked for global perturbations that could alter the properties of all network elements. We sought guidance from the natural biology of the wild-caught animals we study, the crab, *Cancer borealis*. We noted that in their natural environments crabs need to deal with fluctuations in temperature, pH, salinity, and oxygen. Consequently, more than 15 years ago, we started to study the effects of temperature on the stomatogastric nervous system, with the goal of determining the extent to which animals expressing quantitatively distinct solutions to generating circuit function could be robust to temperature change.

**Figure 2.**
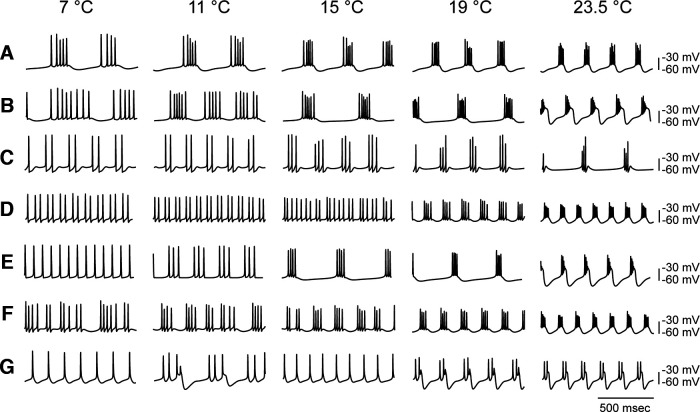
Only some sets of Q10s produce temperature resilient solutions. ***A***, Bursting neuron that maintains its burst over temperature range. ***B–G***, Other sets of conductance Q10s do not produce temperature resilient constant behavior. From Tang et al. (2010).

This is a nontrivial problem, as all biological molecules, including all proteins, are inherently temperature dependent, as their structures are altered by temperature changes. A crude measure of expressing the exponential temperature-dependence of a biological process is the Q10. As a first approximation, the Q10 is the change in a biological process that occurs over a range of 10°C. While many biological processes have Q10s between 1.5 and 4, the Q10 is a function of the structure of the protein, and therefore proteins that usually work together can have quite different Q10s. The significance of this for neuronal excitability is that the temporal relationships among the dynamics of the opening and closing of ion channels that are important for all aspects of neuronal excitability and signaling may get disrupted, as classes of ion channel proteins differentially change as a function of temperature. This is illustrated in the example in Figure 2, which shows a model bursting neuron that maintains its activity over a large temperature range, and a number of other models that do not (Tang et al., 2010). Despite the fact that multiple sets of conductances can produce similar behavior, it turns out that random searches of conductances only rarely find Q10 sets that are temperature robust (Caplan et al., 2014; O’Leary and Marder, 2016; Alonso and Marder, 2020). Thus, evolution has found sets of correlated conductance expression that function together to maintain the dynamics of neuronal and network activity (Tang et al., 2010; O’Leary and Marder, 2016; Alonso and Marder, 2020).

**Figure 3.**
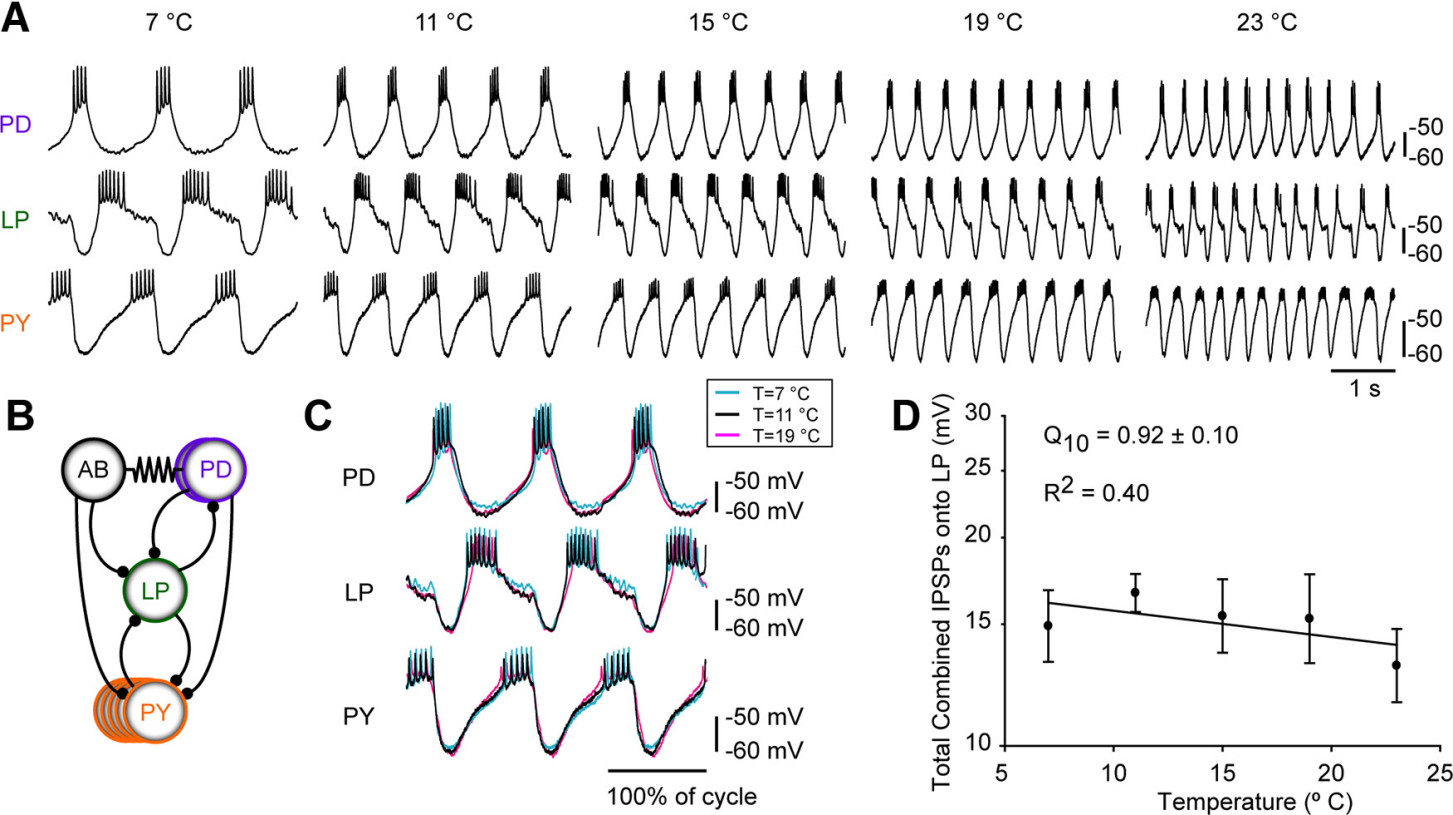
The *C. borealis* triphasic pyloric rhythm is maintained over a large temperature range. ***A***, Simultaneous intracellular recordings from the PD, LP, and PY neurons at temperatures from 7°C to 23°C. Frequency increases but the alternating pattern of activity persists. ***B***, Connectivity diagram of the pyloric rhythm. ***C***, Superposition of the traces at different temperatures by scaling time reveals that the waveforms are preserved. ***D***, Total synaptic input to the LP neuron as a function of temperature. From Tang et al. (2010).

An example of the success of biological systems in maintaining reliable and robust circuit performance as temperature changes can be seen in Figure 3. Simultaneous intracellular recordings of the PD (Pyloric Dilator), LP (Lateral Pyloric), and PY (Pyloric) neurons (Fig. 3*A*) illustrate that the triphasic pyloric rhythm in *C. borealis* is maintained as the temperature is raised 16°C from 7°C to 23°C (Tang et al., 2010). Similar in broad strokes but different in specific details are results from other species, raised in other habitats (Stein et al., 2023).

The remarkable extent to which the attributes of the pyloric rhythm in *C. borealis* are preserved despite the substantial change in frequency is seen in Figure 3*C*, in which the traces were scaled by normalizing to the period. These sorts of data, showing changes in pyloric rhythm frequency while maintaining the other attributes of the rhythm, are seen in recordings from virtually all *C. borealis*, despite the fact that they differ in the specific sets of network conductances that they display (Schulz et al., 2006; Tang et al., 2010).

**Figure 4.**
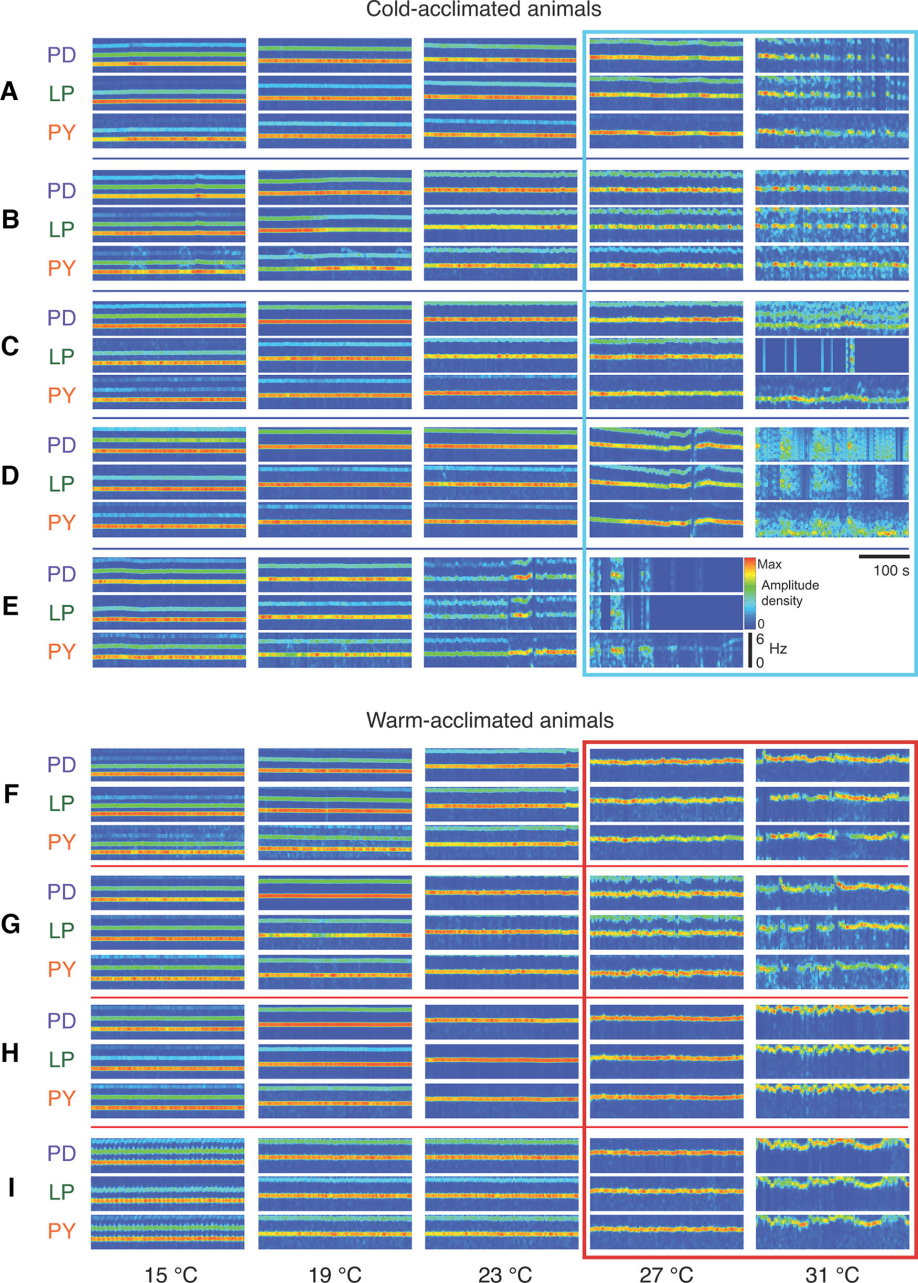
Spectrograms of PD, LP, and PY neuron activity as a function of temperature from cold-acclimated and warm-acclimated animals. The spectrograms are color coded for 0–6 Hz and show 300 s. ***A–E***, Data from five cold-acclimated animals showing crashes at high temperatures. ***F–I***, Data from four warm acclimated animals. Spectrograms are plotted for each neuron, and show that at low temperatures the rhythms are stable, but as the temperature was increased the rhythms become less regular. Importantly, the dynamics of the irregular rhythms are different for each animal. From Tang et al. (2012).

Nonetheless, the effects of these disparate sets of conductances across animals are seen in response to higher temperatures that elicit “crashes” or loss of function (Tang et al., 2010, 2012; Rinberg et al., 2013). Figure 4 shows spectrograms of three neurons of the pyloric rhythm at different temperatures. These spectrograms show reliable and consistent pyloric rhythms at lower temperatures, but as the temperature was increased, the spectrograms became less regular, and then showed patterns of disrupted activity. The dynamics of the disrupted activity are different in each animal, as would be predicted if they have different underlying conductances. Figure 4 also shows that three to four weeks of acclimation at warm temperatures led to more robust rhythms at higher temperatures, and seemed to elevate the crash points.

**Figure 5.**
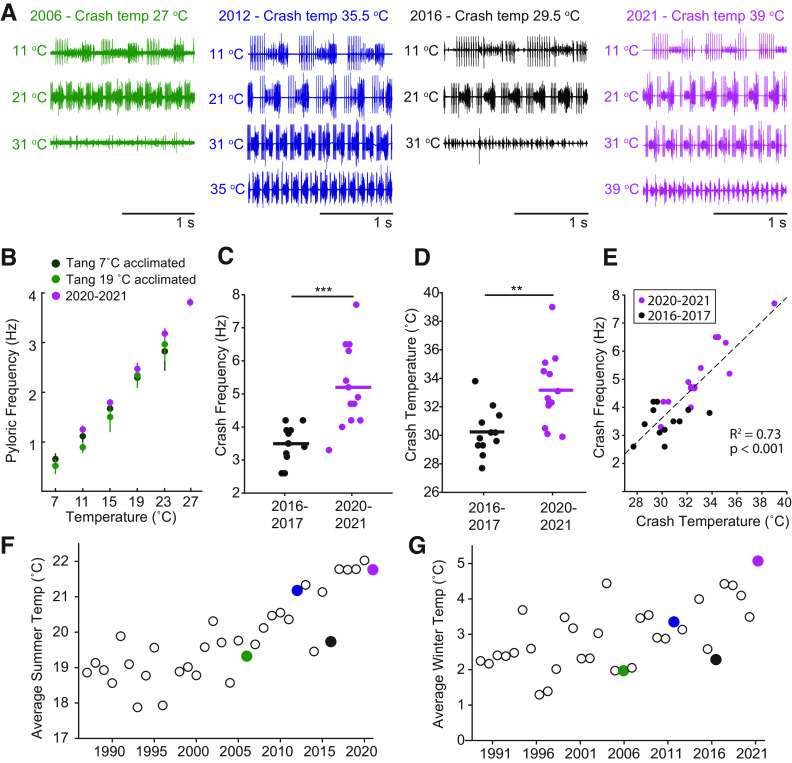
Effect of climate change on pyloric rhythm crashes. ***A***, Extracellular recordings of the triphasic pyloric rhythms at the designated temperatures from the years indicated. Note that in 2006 and 2016 the preparations had completely lost activity by 31°C, but in 2012 and 2021, the rhythms were robust at 31°C and still present at considerably higher temperatures. ***B–E***, Statistics of crash temperatures at different years. ***F***, ***G***, Average water temperatures from a NOAA buoy 16 miles off the coast of Boston. Colored dots are correlated with the data shown in ***A***. From Marder and Rue (2021).

Over the years, we have noticed that, while all animals behave consistently and predictably at lower temperatures, there were yearly changes in the crash temperatures, that were associated with changes in the ocean temperature. This is seen in data that we mined from our notebooks from the years of 2006–2021 (Marder and Rue, 2021), and presented in Figure 5. In 2006 and 2016, the preparations had completely lost activity by 31°C, but in 2012 and 2021, the rhythms were robust at 31°C and still present at considerably higher temperatures. The average ocean water temperatures in 2012 and 2021 were elevated in comparison to those in 2006 and 2016, and the intertidal water temperatures were even more acutely affected than those further off-shore, near to the National Oceanic and Atmospheric Administration (NOAA) buoy that provided these values.

There are several points to be made from the data presented in Figure 5. First, not surprisingly, it is clear that significant acclimation ensues from long-term water temperature fluctuations, and these affect the temperature resilience of crabs for long periods of time. It is notable that the elevation of the crash temperatures in wild-caught animals after months of average ocean temperature elevations was more extreme than what we produced in three to four weeks of acclimation in laboratory tanks. It will be interesting to see whether the natural fluctuations that animals experience in the ocean are important in determining the extent of acclimation that they demonstrate. A new study compares thermal acclimation and habitat-dependent effects of two species of crabs that are showing invasive, habitat expansions. Notably, these species show long-term acclimation to their habitat temperatures, undoubtedly abetting their invasions into novel territories (Stein et al., 2023).

Second, at 11°C, the temperature we routinely use as control temperatures in the laboratory, all of the animals appear “normal.” Nonetheless, these normal activity patterns are hiding cryptic changes that are only revealed in response to subsequent, extreme, perturbations. In an unrelated set of experiments, we also found that high potassium treatments resulted in cryptic changes that last for many hours, and again, are only visible when the preparation is subsequently challenged (Rue et al., 2022; Alonso et al., 2023).

## The Relevance to Human Health

Because humans, like other mammals, have mechanisms to regulate their body temperature, it is often assumed that these mechanisms will protect the brain against all environmental temperature extremes. But, while warm-blooded animals do attempt to maintain approximately constant body temperatures, during fever, exercise, and environmental temperature extremes, core body temperatures do change. In particular, brain and nervous system temperatures increase in response to extreme heat. Even healthy individuals may suffer heat stroke, there are increasing numbers of heat-related deaths (Bouchama et al., 2023), and numerous molecular changes are associated with heat stroke in humans (Bouchama, et al., 2023).

People with compromised nervous systems can be more susceptible to high temperatures than individuals with healthier brains. Specifically, many seizure-prone individuals or people with multiple sclerosis are negatively impacted by temperatures that are without obvious negative consequences for most healthy individuals (Peters et al., 2016; Christogianni et al., 2018). It is easy to speculate that people who suffer from these disorders are effectively closer to circuit “crashes” analogous to those triggered in crabs in elevated temperature. That is to say, many of the mechanisms that protect against circuit instability are likely to be partially compromised in many neurologic disorders. The negative effects of these changes may be cryptic or hidden until the temperature stress. Similarly, we can speculate that people who suffer from posttraumatic stress disorders may be closer to circuit crashes, and seemingly without symptoms until the environmental stress that reveals the presence of the prior changes in brain circuitry.

## Individuals, Populations, and Resilience

We are today challenging humans and animals in new ways. It is always tempting to measure the resilience of population means measured on the basis of large numbers. Indeed, most therapeutic decisions come after clinical trials that depend on a treatment being, on average, better than other alternatives. However, it should go without saying, that both the deleterious effects of climate and therapeutic advances may often not be seen when assessing mean data. Instead, we will require new ways to discover the specific attributes that make individuals, with their individual sets of circuit functions, less resilient to the challenges of environmental and climate change.

The failures of averaging do not obviate the need for reliable statistical measures. Nonetheless, we are all *n* = 1 in regard to our individual histories. For example, many years ago my mother recovered completely from an illness which was thought to be fatal in >99% of people of her age, and sadly, too many of us suffer from relatively unusual or rare ailments. But, we have to live with our reliance on pooled data while understanding that any one of us may be on the tail of a statistical distribution that makes received wisdom not predictive of our unique sets of futures. And I stare out my window at the gray and gloomy Boston harbor and wonder how the crabs and lobsters hidden from me below the harbor’s surface are navigating their disrupted environments, and whether we or they will be here in 50 years.

## Synthesis

Reviewing Editor: Christophe Bernard, INSERM & Institut de Neurosciences des Systèmes Decisions are customarily a result of the Reviewing Editor and the peer reviewers coming together and discussing their recommendations until a consensus is reached. When revisions are invited, a fact-based synthesis statement explaining their decision and outlining what is needed to prepare a revision will be listed below. The following reviewer(s) agreed to reveal their identity: NONE.

No comment
